# Laypersons’ perception of common cold and influenza prevention—a qualitative study in Austria, Belgium and Croatia

**DOI:** 10.1080/13814788.2019.1645831

**Published:** 2019-08-21

**Authors:** Goranka Petricek, Kathryn Hoffmann, Anna Vandenbroucke, Asja Cosic Divjak, Elisabeth Anne-Sophie Mayrhuber, Wim Peersman

**Affiliations:** a Department of Family Medicine, ‘Andrija Štampar’ School of Public Health, School of Medicine, University of Zagreb, Zagreb, Croatia;; b Family Medicine Office, ‘Zagreb Centar’ Health Center, Zagreb, Croatia;; c Department of General Practice and Family Medicine, Center for Public Health, Medical University of Vienna, Vienna, Austria;; d Department of Public Health and Primary Care, Ghent University, Ghent, Belgium;; e Research Group Social and Community Work, Odisee University College, Brussels, Belgium;; f Department of Rehabilitation Sciences, Ghent University, Ghent, Belgium

**Keywords:** Influenza, common cold, laypersons, prevention, qualitative study

## Abstract

**Background:** Common cold and influenza result in an increased number of primary care consultations, significant work/school absences and cause a socio-economic burden. Laypeople’s perceptions and knowledge regarding common cold and influenza prevention is poorly understood and under-researched.

**Objectives:** Our study explores laypeople’s knowledge of prevention of common cold and influenza across three European countries. Furthermore, it investigates if there is any distinction between prevention activities focussing on reasons impacting the attitude towards influenza vaccination as well as investigating cross-country variation.

**Methods:** In total, 85 semi-structured individual interviews were performed across three European countries (Austria *n* = 31, Belgium *n* = 30, Croatia *n* = 24). Qualitative thematic content analysis was performed.

**Results:** Most participants across all three countries made no distinction between the prevention of the common cold and influenza and referenced the same preventative measures for both conditions. They mainly expressed negative attitudes towards influenza vaccination possibly effective but only intended for high-risk groups (bedridden/older people, chronic patients or health workers). There were very few cross-country differences in results.

**Conclusion:** The perception of health risk of contracting influenza and a primary healthcare physicians’ recommendation played an important role in shaping participants’ decisions towards vaccination. Primary healthcare physicians are invited to assess and if necessary adjust inappropriate prevention behaviour through their everyday patient consultations as well as add to the knowledge about influenza severity and influenza vaccination benefits to their patients.

KEY MESSAGES

- Most participants do not distinguish between the prevention measures against common cold and influenza.
- They declared mostly negative attitudes towards influenza vaccination, intended only for high-risk groups.
- The perception of health risk of contracting influenza and a primary healthcare physicians’ recommendation played an important role in shaping participants’ decisions towards vaccination.

## Introduction

Worldwide, acute viral respiratory tract infections are the most common human illnesses. The common cold is a mild, self-limiting infectious disease of the upper respiratory tract caused by a variety of viruses [1], which have been estimated to cause 34% of all respiratory illnesses [2], with an incidence in adults three to five times and in children up to 10 times a year [3]. Influenza (‘the flu’), is a more serious acute respiratory tract infection, primarily caused by different serotypes of influenza viruses [1], it presents itself globally with epidemic outbreaks every two to three years, with a yearly incidence of up to 20%. Consequently, common cold and influenza result in an increased number of primary care consultations and significant work/school absence representing a significant public health issue, which raises the question of prevention importance [1,4–6]. However, studies to date focused more on their clinical aspects and treatment possibilities while only a few studies focused on prevention, emphasizing the effect of certain physical interventions, over the counter drugs and influenza vaccination [3,7–10].

This is the second paper that draws on a qualitative study by our group, which investigated how individuals across Europe, namely in Austria, Belgium and Croatia perceive common cold and influenza symptoms and prevention and their differences [11]. Countries were selected because of their locations in different geographical regions of Europe to explore cross-country variations thoroughly [11]. In our first paper, we elaborated on layperson’s understanding of common cold and influenza symptoms, pathogenesis and differences between those diseases across three European countries, which, according to analysis, was fairly good although explanations integrated certain misconceptions such as misinterpretation of fever, disease continuums or diverse onset ideas [11].

In the current paper, we aimed to explore layperson’s knowledge about prevention of common cold and influenza, investigate if there is any distinction between prevention activities against those diseases. Special accent was placed on participants’ reasons impacting their attitude towards influenza vaccination as well as investigating cross-country variations. This specific layperson’s prevention knowledge, still poorly understood and under-researched, is important for primary healthcare physicians in the provision of person-centred care.

## Methods

### Study design

This study is designed as a qualitative research study including participants from urban and suburban areas of three European regions: eastern Austria, Flanders (Dutch-speaking area of Belgium) and Zagreb in Croatia.

Semi-structured individual interviews were performed using an interview guide containing open-ended questions based on existing literature [11], developed by the second author (KH) and translated to each countries respective language (Supplementary material). In line with the research question, a qualitative approach is particularly suitable for gaining explanatory and meaningful explanation to the study aims [12].

### Ethics

approved the study.

### Selection of study subjects

To recruit participants the purposive sampling was applied, following predetermined inclusion criteria—at least 18 years old, physically and psychologically able to participate in the study, able to communicate in the respective country language and live in Vienna or lower Austria (Austria), Flanders (Belgium) and urban or suburban area of Zagreb (Croatia)—and exclusion criteria—participants who worked in a health-related field, and in Belgium if their family studied for or worked in a health-related field. Sampling approaches somewhat differed between the countries: in Austria and Belgium, the interviewers recruited participants from the general population according to inclusion and exclusion criteria. Approximately half of the Austrian participants came from the urban part of Vienna and the others from rural lower Austria (31 participants). All Belgian participants were living in Flanders (30 participants). In Croatia, the study was conducted in a general practice setting: six general practitioners (GPs) were selected from the health centre ‘Zagreb-Centar’ (three from urban and three from the suburban areas of Zagreb) to recruit the patients. Each GP recruited four patients from their list according to the inclusion and exclusion criteria (24 participants). The data collection was conducted in the period from November 2013 to June 2014 in Austria, February 2016 to May 2016 in Flanders and from March to July 2016 in Croatia.

First, participants were contacted and informed about the purpose and design of the study and second, invited to participate (in Austria and Belgium by the interviewer, in Croatia by the GP). Only one patient from Croatia refused due to lack of time and was replaced by another patient from the respective GP’s patient list. Those willing to participate received an official letter of request, and signed a written consent form before participation.

### Data collection

The semi-structured, individual interviews were performed and transcribed verbatim. Two Austrian medical diploma students (CS and FK), two Belgium master’s students in Health Education and Health Promotion (NvdK and AV) and a Croatian family medicine vocational trainee (ACD) conducted the interviews. The five were trained in qualitative methods and supervised by their mentors (KH, WP, GP). All participants filled in a small quantitative questionnaire to collect sociodemographic data (gender, age and level of education). All recorded interviews held in countries native languages (German, Dutch or Croatian) were conducted at a place of the participant’s choice and lasted from 15–45 min. All 85 transcripts met Kvale’s quality assurance criteria and they were used for the analysis [13].

### Data analysis

The transcribed data were, as described by Pope et al., explored inductively using content analysis in accordance to the research questions (first open coding, defining as many codes as needed to describe all aspects of the content, second the codes were categorized to create themes and sub themes, all leading to an explanation) [14]. At about the nineteenth/twentieth interview in every country it was felt that the emerging explanation was sufficiently developed: content saturation had been reached [12]. This also confirmed sufficient sample size.

This paper presents the patients’ reflections and explanations on questions in the interview guide: ‘how do you protect yourself against common cold and influenza?’ In some cases, the following supplementary questions were used: ‘did you take any home remedy or medication? Where did you get it from? Did you get influenza vaccination?’

A qualitative content analysis coding was performed by one researcher per country (EAM, AV and ACD) according to the research questions by using the Atlas.ti (ATLAS.ti Scientific Software Development GmbH, Berlin, Germany) or NVivo (QSR International, Melbourne, Australia) analytic tools [12,14,15]. Subsequently, the codes were summarized and discussed with other authors of this paper within the country (for Austria: EM, KH; for Belgium: WP, AV; for Croatia: ACD, GP, and: ZOA, VC) and then those national results were merged. As the starting point for the discussion of the transnational results, the Austrian results were used. Final results represent a product of a thorough discussion between researchers from each participating country. Participants’ answers were translated into English and are presented as quotes in the results section.

## Results

### Participants’ characteristics

In this study, 40 of the 85 participants recruited were male (40/85). All participants were between the ages of 18 and 83, with different levels of education as well as different areas of residence (Table 1). In Belgium, interviews were conducted with a more significant number of people with higher education background, which was, however, not reflected in the results. All participants declared personal experience with both common cold and influenza (85/85).

**Table 1.** Participants’ characteristics.

Age, years	Austria	Belgium	Croatia
18–30	6	14	9
31–45	6	4	2
46–60	7	9	4
61–75	6	2	7
76+	6	1	2
Gender			
Female	19	16	10
Male	12	14	14
Level of education			
Higher education	2	16	11
Elementary/high school	29	14	13
Influenza vaccination in the year of interviewing			
Yes	5	6	5
No	26	24	19

### Prevention of the common cold (Box 1)

Most participants expressed awareness of strategies to prevent common cold. Among preventative measures they accentuated reinforcing individuals’ immune system by leading a healthy lifestyle—eating vitamin-rich foods, mostly containing vitamin C, taking various vitamin supplements, consuming other products based on plants and herbs (tea, honey, ginger) and regular exercise. Only one participant in Belgium mentioned taking analgesic and antipyretic medication for prevention purposes. Tending to personal hygiene (hand-washing, avoiding direct contact with sick people or contaminated objects, frequently ventilating and cleaning rooms they live in) were strongly pointed out too as well as adequate weather wear along with avoiding environmental factors such as drafts and cold temperatures. One category noted only in Austria and Croatia was avoiding larger groups of people, especially concerning public transport. In Belgium, several participants mentioned large groups (in small rooms) as a risk factor but they did not avoid those situations as a strategy against the common cold.

**Box 1:** Prevention of the common cold

Prevention of infection by hygiene measures	Avoiding contact with sick people/contaminated objects	‘Maybe I try to avoid contact with persons who show clear symptoms of flu-like diseases during these seasons.’ (A9) ‘I do not touch the things, the door handles and such, especially in public spaces. ‘ (A6) ‘Pay attention to what you drink from and with whom you come into contact.’ (B18) ‘I try not to get in contact with someone sick, if someone home is sick, we don’t really hug and kiss.’ (C7)
	Hand hygiene	‘Maybe I try, during these time periods, to avoid skin contact to surfaces that many people touch, and I possibly try to wash hands more often. If I, after I touched a surface such as, i.e. door handles and switches and other, I will try not to touch my face…’ (A19) ‘I wash my hands.’ (B6) ‘I wash my hands often…’ (C7)
	Ventilate/clean rooms	‘I am convinced that if you get it, that it is in the air, so that you have to open everything every now and then.’ (B12) ‘Cleaning the apartment more often…’ (C12)
	Avoiding larger groups of people	‘You should not attend larger crowds.’ (A21) ‘I generally avoid going to crowded places, more or less.’ (C19)
Strengthening the immune system	Healthy lifestyle	‘… during the autumn I pay attention that I eat a lot of vitamins, that I take in fruit sufficiently, which is not always easy.’ (A3) ‘I regularly go to the gym and also regularly to the sauna afterwards.’ (A29) ‘Try to live a healthy life. Sufficient sleep, sufficient exercise and good food.’ (B16) ‘… I think person’s general fitness, body constitution, maintaining physical fitness, in my opinion that is most important.’ (C4)
	Products based on plants/herbs	‘Oranges with honey, tea with honey, yes.’ (A23) ‘… drinking tea, and honey.’ (B7) ‘I heard about ginger, honey and lemon combination so I took that once…’ (C16)
	Vitamin supplements	‘… then I take Echinacea pastilles.’ (B1) ‘… sometimes I use vitamin pills.’ (B2) ‘… most often I prepare myself a lemonade at home, or I buy effervescent tablets with vitamin C.’ (C3)
Adequate weather wear, avoiding drafts/cold temperatures		‘How I protect myself … by not wearing light clothing when I go outside, when it’s cold or that I avoid draft.’ (A10) ‘… take into account that if I have washed my hair, that it is well dry before I go outside.’ (B15) ‘I dress according to the weather conditions, and I take care not to get in weather-temperature ‘traps’.’ (C10)
Don’t do anything		‘Actually I hardly change anything. In my opinion I have a relatively balanced diet, there may be possibilities for improvement, but I don’t follow a really super healthy diet, as I would wish for, that is why I don’t do much for it.’ (A11) ‘Either you are lucky or you are unlucky … well I don’t think you can protect yourself (note: from falling ill).’ (A31) ‘I seriously don’t need to protect myself … up till now my body was able to fight those things on its own.’ (C17)

In contrast to abovementioned measures, some of the participants considered preventative measures regarding common cold needless, assuming their body is capable of defending itself without additional support. Additionally, some of them stated there is no way you can prevent getting a cold because it is just something that happens in autumn and winter: ‘if it has to come, it will come’ (B5).

### Prevention of influenza (Box 2)

Most participants have not made a distinction between the prevention of common cold and the prevention of influenza. They referenced the same preventative measures for influenza as for the common cold. Several participants even explained it not as preventing common cold or influenza but as prevention of getting ill or being infected. Some of the participants stated again they did not do anything regarding the prevention of influenza.

**Box 2:** Prevention of the influenza

Prevention of infection by hygiene measures	Avoiding contact with sick people contaminated objects	‘What plays a role in my case maybe, that I got the flu less frequently was that I went with the car to work, therefore I hardly had contact … in public transport.’ (A29) ‘I avoid having contact with people I know have the flu, that’s for sure.’ (C2) ‘I do not use cutlery from someone with the flu, or drink from his glass.’ (B25)
	Hand hygiene	‘… I wash my hands more often, after buying groceries, or after I went somewhere.’ (A2) ‘When it’s flu season, I mostly wash my hands, when I’m at the doctors’ or something like that, I always wash my hands. I wouldn’t want to get infected, so I wash my hands because I know I’ll touch my face later.’ (C2)
	Ventilate/clean rooms	‘… it is important that you always ventilate everything well. If someone comes to us and I know they are sick, when they are gone, I will open everything for a quarter of an hour.’ (B10)
	Avoiding larger groups of people	‘I mean, it’s like they say, don’t go into enclosed spaces where there are people, as much as you can stick to that, you can easily catch it in a streetcar, or at work.’ (C16)
Strengthening the immune system	Healthy lifestyle	‘Well, the same way, I guess, like against the cold. Diverse nutrition, sports life….’ (C15) ‘Eating enough fruit to maintain your immune system, that’s the key I think, and yes, enough sleep.’ (B17)
	Vitamin supplements	‘… by taking some vitamin C….’ (B28)
	Taking other products to strengthen the immune system	‘I take homeopathic grains, with influenenzinum, which I always have, against the flu. I take them first every week, and then once a month as long as the flu is present.’ (B19)
Adequate weather wear, avoiding drafts/cold temperatures		‘I try to cover myself, a scarf or something.’ (B23) ‘Well, you should go about your normal activities, dress appropriately, so you’re comfortable, not too hot or too cold. That’s how I prevent the flu.’ (C10)
Vaccination		‘… an annual flu shot at the doctor’s office.’ (B14) ‘I get vaccinated against the flu … must be 15 years now … because I’m got other chronic conditions.’ (C5)
Don’t do anything		‘Independent of the influenza season I wash my hands often, not only when it’s the season.’ (A11) ‘I don’t do anything to protect myself, not even vaccination.’ (C20)

Again, slight differences between countries were observed, for instance, few participants in Belgium mentioned taking vitamin supplements for influenza prevention.

### Attitudes towards influenza vaccination (Box 3)

Although most of the participants were well aware of the possibility for influenza vaccination, only a small number received an influenza vaccination (16/85).

**Box 3:** Attitude towards influenza vaccination.

Reasons for vaccination	Fear of influenza/complications	‘I did not get influenza vaccination for so many years, but I took it for this year because well, and there is maybe this point in time, because I am retired for several years now, I now suffer again more often from the flu, while maybe that is the case when one becomes older….’ (A29) ‘If you are sick then it is more difficult to cure [without a vaccine]. Your resistance reduced. ‘ (B14) ‘I have to, since I had that bacterial pneumonia, I get vaccinated every year. I’m afraid of the flu, absolutely afraid. I think you should get vaccinated. I didn’t get it once since I started vaccinating myself. Not even a cold. Nothing.’ (C1)
	High risk for influenza (chronic patients, older people, health workers)	‘After I suffered my second stroke, the mister doctor said vaccination—every year. (…) And this is what I did and since then I have not had the flu and it has been 10 years since the second stroke.’ (A28) ‘The doctors then said that I was a risk patient for the flu because I had asthma and since then I started doing it every year.’ (B6) ‘I’ve been getting vaccinated for years now, because I’m a chronic patient.’ (C5)
	Health professionals recommendation	‘I got it prescribed [note: because of COPD].’ (A8) ‘…the doctor was a supporter of that.’ (B11) ‘But in terms of prevention, especially if you’re retired and older like I am, then it’s recommended to get vaccinated and I do it every year.’ (C10)
Reasons against vaccination	Perceived low influenza susceptibility, or low impact of influenza	‘I am strictly against it because I hear from everyone who got the influenza vaccination that they contracted something [note: an illness] and I didn’t.’ (A5) ‘I do not think that’s worth the effort. I cannot remember that I had the flu.’ (B7) ‘I think I’m not susceptible to flu and I don’t have to get vaccinated.’ (C4)
	Vaccination only for risk groups	‘I already heard about it and I have talked to my GP and he said he does not vaccinate people under 60 years. Because, he says, if you are not really ill or have a chronic disease then the body is strong enough to overcome the flu (…) I think the body should learn to fight (…).’ (A25) ‘If you are healthy, then you should not let yourself be injected with a disease-something.’ (B1) ‘I heard there were vaccines, but it’s mostly for older people and people who work in healthcare, they are much more in contact with persons that have the flu.’ (C9)
	Perceived vaccine ineffectiveness	‘My sister for example, she obtains the influenza vaccination every year, every year she has a heavy flu, every year a heavy flu, I say, why do you get the vaccination, what for? It has no use the vaccination. The best is to ‘sweat through’ it.’ (A13) ‘They told on the news that it does not work well, or that it is only one in two or something.’ (B2) ‘If you are vaccinated against the flu, you will get another variant.’ (B8) ‘Ultimately, I believe vaccines against viral infections to be quite ineffective. I mean, even if we get vaccinated against one virus, there is some kind of other strain that can cause the same consequences, and we never got vaccinated against it. I think it’s better to boost your immune system, than get vaccinated.’ (C13)
	Fear of vaccine side effects	‘I received the influenza vaccination once and then I got the symptoms of the flu and since then I don’t get it no more.’ (A16) ‘You are then vaccinated and you also hear from some people that they are sick of that vaccination.’ (B12) ‘In the US, the vaccines used here are banned because there is too much lead and mercury in them.’ (B21) ‘I’m not sure if I could achieve the opposite effect, by getting vaccinated.’ (C4)
	Contraindications to vaccination	‘I don’t get vaccinated because I am not allowed [note: suffers from a demyelinating disease]… Once I received the influenza vaccination, I was 17 … Then I was ill for three months.’ (A30)
	It is not a priority	‘Every year when I have the flu, I say: “shit, I really have to do that next year [Taking a vaccine]”, but I don’t. (…). It is a little bit human for sure, when you are sick, you say “I should have done it”, but then you forget. I’m called every year, but I have to go to X (city) for the vaccine, and I do not do that then.’ (B3) ‘To be honest, I make an appointment (to be vaccinated), but always something gets in the way so I never make it … usually something meaningless….’ (C3)
	Greater trust in homeopathic	‘Yes, what do they put in those syringes. (…) Just give me what nature gives and I think that’s worth my trust.’ (B19)
Reasons to get vaccinated it in the future	Advice of their doctor	‘… but then my doctor has to say “now it is needed”.’ (B9)
	Belonging to a risk group/increased risk of influenza	‘Yes, if you are old and have no resistance anymore.’ (B10) ‘Maybe I will when I’m sixty or something like that when it will be more critical than now, but so far I see no need.’ (C9)
	Protecting other people	‘If someone can have a disadvantage, I will do it immediately.’ (B13) ‘Probably when I have kids, their health will be important to me so I’ll probably start thinking of myself as well. ‘ (C12)
	Having influenza/contact with influenza frequently	‘… but I intend to because I think prevention is really important. What I’ve done so far was some kind of lottery because I’m frequently among people that have flu….’ (C3) ‘During my internship at a primary school, I talked about it and I heard that many teachers do so. So then I have thought about it that maybe I would do that later.’ (B2)
	Not becoming sick during an important moment	‘I would take a flu vaccine if it is an important period that is accompanied by a flu epidemic.’ (B4)

The main reasons among participants against influenza vaccination were perception of being at low risk for influenza, impression that vaccination is necessary only for risk groups (bedridden/older people, chronic patients or health workers), debatable efficiency of the vaccine as well as fear of vaccine side effects. One Austrian participant explained that she could not get vaccinated due to her pre-existing chronic illness. Some participants, in Belgium only, expressed greater trust in homoeopathic medicine and distrust toward vaccination. In addition, in Belgium, some participants emphasized the self-limiting character of influenza as a reason not to get vaccinated. Conversely, among the participants that were vaccinated, reasons for supporting vaccination were recommendation of health professionals, particularly primary healthcare physicians, fear of influenza and possible complications, and self-perception of being at risk to contract influenza (e.g. chronic patients, older people). Besides, participants in Belgium that were vaccinated in the year of interviewing, stated that they would recommend this preventive measure to everybody, regardless of age and/or being part of any risk group.

In Austria, participants declared no intention of getting influenza vaccination in the future. Whilst some of them in Belgium and Croatia stated the following reasons for getting vaccinated in the future, belonging to a risk group (getting older), protecting other people and change of circumstances (having influenza/more frequently contact with influenza infected people). In Belgium, only some participants considered vaccination to prevent becoming sick during a crucial moment (e.g. exams, important period at work).

## Discussion

### Main findings

This study provides the laypersons’ perceptions of common cold and influenza infection prevention across three European countries: Austria, Belgium and Croatia. Although not always explicitly declaring that common cold and influenza are contagious diseases, analysis of our participants’ experiences have clearly shown that most of them possess a fairly accurate perception of both diseases transfer, and consequently basic infection control practices. Albeit, results show that participants across the three countries mostly have not made a distinction between the prevention of common cold and influenza and referenced only general preventative measures for both conditions. Most participants neglected vaccination as a possible preventative measure against influenza expressing negative attitudes towards vaccination and considering it possibly effective but only intended for high-risk groups (bedridden/older people, chronic patients or health workers). There were very few cross-country differences in results.

### Strengths and limitations

The strength of this study is its qualitative design, often used for an in-depth understanding of participants’ beliefs and attitudes pertaining to topics of investigation. Although this study took place in three different European countries, the analysis showed high homogeneity of the main representation dimensions, so it may highlight some issues relevant to the general population’s behaviour related to influenza and common cold prevention.

One of the weaknesses was the sampling of patients. We chose respondents who wanted to participate and were mobile, which excluded those who often avoid company, who are introverted, do not want to talk about themselves, or are very sick or frail. Maybe that group of respondents would have other ideas about the topics of the interview. Nevertheless, our participants came from a wide range of socio-economic backgrounds, but still we cannot assume that other themes would not emerge in different localities or cultural groups. Furthermore, since the study data is dependent on participants reporting their previous experiences, it is also possible recall bias occurred.

The second limitation is the difference between sampling periods: influenza season 2013/2014 in Austria and influenza season 2015/2016 in the other two countries. These two periods could influence the results across the three countries. However, the effect of different sampling period is minor due to similarities in severity of mentioned influenza seasons. Moreover, milder influenza seasons could have repercussions on the layperson’s perception and affect the percentage of vaccinated participants. This should also be considered when interpreting results of this study.

### Interpretation in relation to existing literature

Most participants in this study expressed awareness of general prevention measures of the common cold and influenza. Some participants stated to refrain from particular behavioural prevention measures, arguing that they considered themselves as healthy and not at risk of contracting infectious diseases. Likewise, investigating influenza prevention behaviour, Seale et al., found an increase of preventive behaviours only in Australian respondents who considered themselves at risk. Whilst Gilles et al., revealed that the recognition of the threat served as a predictor of the perceived respiratory infection prevention measures efficacy in Swiss respondents [16,17].

Despite most of our participants’ awareness of influenza vaccination possibility, only a minority employed that practice. These findings are highly consistent with the health belief model (HBM) and the social cognitive theory (SCT), both theoretical models often used to understand patient behaviour regarding illness prevention [18,19]. As suggested by the HBM, our participants’ positive attitude towards vaccination correlated with positive advice of health professionals, perceived high influenza complication severity as well as influenza susceptibility (chronic patients, older people) [18], also following other studies results [9,20–26].

In further correlation with HBM and SCT, our participants’ negative attitudes towards vaccination corresponded with perceived low personal risk for contagion/low illness severity, perception of debatable efficiency of the vaccine or negative opinions about the vaccine consequences similarly to other studies: ‘The vaccine does not work’ [9,27–29]; ‘I never get the flu/I am healthy’ as well as ‘The vaccine causes the flu’ [4,9,29,30].

The phenomenon of perceived influenza susceptibility, described by our participants as belonging to a risk group, could be in accordance with the category ‘frail people’ defined by Cedraschi et al., [31]. Interestingly, most participants from both studies did not consider themselves within this category [31]. Similar to our findings, that personal belief of being healthy as well as belief that alternative protective lifestyle (eating healthily and exercising) could confer immunity was emphasized by Rubinstein et al., as a barrier for vaccination [23]. Furthermore, participants in Belgium and Croatia highlighted a possibility of personal risk alteration indicating that with time or change of circumstances, influenza vaccination could be a prospect for them, a notion not found in literature so far.

### Implications for clinical practice, education and further research

A relatively good understanding of general prevention of respiratory infections was found in this study, indicating that it is necessary to invest time in individual patient education regarding influenza vaccination. Having in mind low influenza vaccination rates [32], insufficient vaccination practice expressed by our participants (6% or 15/85 were vaccinated) should direct further interventions. The leading reasons among participants against influenza vaccination (perception of being at low risk for influenza, impression that vaccination is necessary only for risk groups and debatable efficiency of the vaccine) confirms the importance of the layperson’s beliefs in their decision to have influenza immunisation but also insufficient knowledge about influenza severity as well influenza country vaccination policy. The perception that the health risk of contracting influenza is low, specifically for people with chronic diseases needs to be challenged by health workers and health education programmes through different media. Moreover, in shaping participants’ decisions towards vaccination, we found that besides a self-perception of being at risk to contract influenza, a primary healthcare physician’s recommendation played an important role. Making healthcare professionals more aware of their influence in shaping participants’ decisions towards vaccination during graduate and postgraduate education as well in continuing professional development, may prompt them to carefully determine and, if necessary, adjust inappropriate prevention behaviour through their everyday patient consultations as well as add to the knowledge about influenza severity and influenza vaccination benefits of the population under their care.

## Conclusion

Most of the study participants expressed a good general prevention understanding and made no distinction between prevention activities against the common cold and influenza. Influenza vaccination was generally only considered necessary for certain risk groups. There were very few cross-country differentiations in results. In conclusion, primary healthcare physicians’ recommendation was recognized as an important facilitator in forming a positive attitude towards influenza vaccination. Practitioners are invited to assess patient’s attitude toward vaccination and, if needed, add to the knowledge about influenza severity as well influenza vaccination benefits possibly affecting a better influenza prevention understanding during their person-centred consultations.

## Supplementary Material

Supplemental data for this article can be accessed here.
