# Supplementary material for: Laypersons’ perception of common cold and influenza prevention—a qualitative study in Austria, Belgium and Croatia
Source: Eur J Gen Pract. 2019 Aug 21;25(4):220–8. doi: 10.1080/13814788.2019.1645831 (PMC6853228; doi:10.1080/13814788.2019.1645831)
Supplement: Supplemental data for this article can be accessed here. [file IGEN_A_1645831_SM5491.doc]

Interview Guide Common cold and Flu

1. Perceptions of the common cold

| *Here the aim is to explore how the respondent perceives a common cold, what it is for her/him.* |
| --- |

How would you describe a common cold?

How do you notice that you have a common cold?

In your opinion, how do you get a common cold?

How often do you get a common cold?

How was it the last time you had a common cold? (Process)

What symptoms did you have and how did they develop?

Are there differences in the common colds you get? (Seasonality, severity)

1. **Treatment of the common cold (home remedies/medical plants and self-medication)**

| *Here the aim is to explore the knowledge the respondent has about the treatment of the common cold, and to find out where she or he has the knowledge from.* |
| --- |

How do you protect yourself against a common cold in general? (Before the disease)

Once you have it what do you do then?

Do you know of any drugs that could help? Do you use any? If yes, when and for how long? Where do you get them from (only one source or different sources)? If no, why not?

What home remedies/medical plants do you know against the common cold (very generally)? (**freelisting**, when finished ask if she knows any more)

(take the items of the freelist and ask for every item) : how do you prepare that home remedy/medical herb for a common cold? What help is it supposed to give?

Where/from whom did you find out about the home remedy/medical herb?

Do you use any home remedies/medical herbs? If yes, which ones and when? Where do you get the home remedy from? Since when do you use them? And why?

How were you treated for the common cold when you were a child?

Where was that?

Who treated you and with what?

1. **Common cold versus influenza**

| *Here the aim is to find out what people know about influenza and how they compare it to the common cold.* |
| --- |

How would you describe a real flu? (Influenza)

Did you have a real flu already?

How was it? How did it begin? How long etc.

What did you do against it?

Did you take medication? Which one? Where do you get it from?

Do you know any home remedy against the real flu?

How do you protect yourself against influenza?

Ask also specifically about vaccination (why yes/no)?
